# Cariogenic Biofilm: Pathology-Related Phenotypes and Targeted Therapy

**DOI:** 10.3390/microorganisms9061311

**Published:** 2021-06-16

**Authors:** Xiuqin Chen, Eric Banan-Mwine Daliri, Akanksha Tyagi, Deog-Hwan Oh

**Affiliations:** Department of Food Science and Biotechnology, College of Agriculture and Life Sciences, Kangwon National University, Chuncheon 200-701, Korea; chenxiuqin0127@kangwon.ac.kr (X.C.); ericdaliri@kangwon.ac.kr (E.B.-M.D.); akanksha@kangwon.ac.kr (A.T.)

**Keywords:** cariogenic niche, interspecies interactions, pathogenic traits, therapeutic targets

## Abstract

The initiation and development of cariogenic (that is, caries-related) biofilms are the result of the disruption of homeostasis in the oral microenvironment. There is a daily accumulation of dental biofilm on the surface of teeth and its matrix of extracellular polymers supports the host in its defense against invading microbes, thus helping to achieve oral microbial homeostasis. However, the homeostasis can be broken down under certain circumstances such as during long-term exposure to a low pH environment which results in the dominance of acidogenic and acid-tolerating species in the dental biofilm and, thus, triggers the shift of harmless biofilm to an acidic one. This work aims to explore microbial diversity and the quorum sensing of dental biofilm and their important contributions to oral health and disease. The complex and multispecies ecosystems of the cariogenic biofilm pose significant challenges for the modulation of the oral microenvironment. Promising treatment strategies are those that target cariogenic niches with high specificity without disrupting the balance of the surrounding oral microbiota. Here, we summarized the recent advances in modulating cariogenic biofilm and/or controlling its pathogenic traits.

## 1. Introduction

Dental caries are a well-established multifactorial biofilm-mediated prevalent chronic oral disease. Poor dietary habits, poor oral hygiene, and caries-associated microorganisms are involved in the formation of the cariogenic ecosystem, which is rich in microbial metabolic acids. Caries-associated microorganisms refer to a restricted subset of species that have caries-related phenotypes and are generally aciduric. As oral microbiology and the science of matrix biology evolves, the pathogenesis of caries become clear. Frequent intake of sugar fuels the accumulation of glycometabolic microorganisms and their by-products such as organic acids and polysaccharides, thereby enhancing the adhesion of microbial cells to the surface of the teeth and creating an acidic pH environment [1]. The demineralization of the localized hard dental tissues is the outcome of acidification of dental biofilm that progresses over time [2]. The environmental changes that occurs in dental biofilm could also affect the metabolic activity of the microorganisms [3,4]. The term cariogenic biofilm can be used to describe the microbial communities that create localized acidic pH niches [5]. Although early studies recognized that cariogenic biofilm is a reason for a net loss of mineral and, thus, may result in a caries lesion [6], outstanding questions related to the pathology-related phenotypes and the diagnosis of cariogenic biofilm are paramount to answer. It is particularly important to explore the cause and process of the transformation from commensal biofilm to cariogenic biofilm.

**Figure 1:**
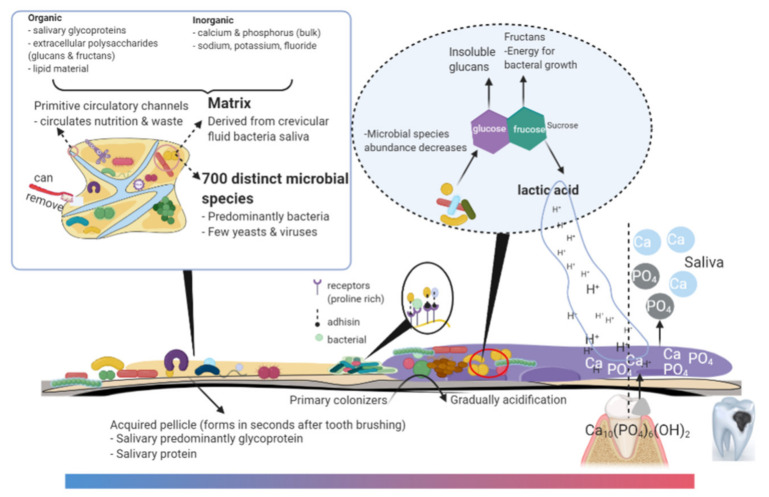
Oral biofilm microenvironment and the trigger of their cariogenicity. Biofilms are present throughout the surface of teeth with a high level of spatial matrix organization and up to 700 distinct microbial species or phylotypes. Primary colonization of microbes on the tooth surface relies on the acquired pellicle and co-adherence with other oral commensals. The ability of microbes to convert sugars into acids is a potent driver for oral biofilms developing into cariogenic biofilms. The acidification of the environment leads to the release of calcium and phosphates into saliva. The illustration was created with Bio-Render.

The structure of dental biofilm is shown in Figure 1. Microbial metabolic activity is the driving force of microbiome composition. The metabolism of microbes in the biofilm interacts to determine the biofilm microenvironment. There is evidence that controlling the consumption of sugars could improve the oral health adolescents [2]. The ability of metabolic sugars to form insoluble glucans associated with the cariogenicity of microorganisms has been reported [7]. With frequent cariogenic factors (high sugar dietary behaviors and/or inappropriate hygiene practices) exposure, the resident plaque microflora shifts to a more cariogenic one and progressively develops into a relatively stable ecosystem. Cariogenic biofilm is an extremely active and complicated ecosystem rich in organic acid. In this review, we discussed the ecological dysbiosis of dental biofilm, their caries-related phenotypes, the current therapeutic targets of cariogenic biofilm, and/or the control of cariogenic biofilm on its pathogenic traits.

## 2. Microbes, Quorum Sensing, and Matrix in Cariogenic Biofilm

### 2.1. Biofilm Microbiota: Opportunistic Pathogens and Commensals

The frequent intake of fermentable carbohydrates that can be metabolized by a specific group of “cariogenic” commensals contribute to lowering the biofilm-pH [8]. As can be observed from previous studies, the definition of cariogenic oral microorganisms is still controversial and their use as a biomarker to determine the risk of caries remains limited. The limitation may be due to the oral microbial communities that are associated with dental caries in different patients and the fact that they are different. Molecular microbiology studies have defined opportunistic caries-associated microbes in the dental biofilm and the pathogenesis of those specific groups could be derived by one or more of the following reasons. Firstly, the microbial groups are selected to be best suited for cariogenic environments [9]. Secondly, antimicrobial metabolites from the microbial groups can reduce the survival of oral commensals and result in the dominance of those groups in the plaque community [10]. Thirdly, the associated stress response can cause a genotypic change which enhances the expression of caries-related phenotypes of those groups [11].

#### 2.1.1. Streptococcus Species

Generally, it is accepted that *Streptococcus* spp. is considered as initial colonizers of dental biofilm and that *Streptococcus mutans* (*S. mutans*) is a common etiologic agent in cariology due to its virulence and ability to metabolize and process sugar carbohydrates [12]. Exoenzymes (Gtfs) of *S. mutans* can use various sugar carbohydrates to produce glucan polymer, which renders the biofilm recalcitrant to antimicrobials and difficult to remove [8]. Some researchers have also evaluated the potential dental caries risk by focusing on the cariogenic bacteria *S. salivarius*, *S. sobrinus*, and *S. parasanguinis* [13]. Early studies led to the proposal that the levels of *S. mutans* in the mouth positively correlated with the degree of dental caries. However, subsequent studies confirmed that caries may occur in the absence of *S. mutans*. While this organism could persist on other surfaces that remained sound, it is confirmed that *S. mutans* alone could not be used to predict carious risk [14]. These findings provided support for caries as a consequence of nonspecific microorganisms. Beyond the Streptococcus species, other species with acidogenic phenotypes play important roles in extending periods of low pH in the biofilms.

#### 2.1.2. Bifidobacterium Species

*Bifidobacterium dentium DSM20436* can metabolize raffinose to a higher extent than *S. mutans*. This can indicate the cariogenicity of bifidobacteria because raffinose is naturally present in healthy foods consumed on a daily basis rather than snacks or sweets [15]. Caries risk cannot be inferred from studies of single species and different organisms display certain significance for the caries attack. Recent studies have shown that there are substantial differences in the composition of the microbiota in biofilms overlying caries lesions.

#### 2.1.3. Lactobacillus Species

According to the study carried by Bourgeois et al., (2017), *Lactobacilli* species were detected in caries-free subjects [16]. However, these species have also been detected to be associated with teeth lesions. The contradictions seen in the results between different studies may be explained by the proportion of each species in the biofilm microbiota. *Lactobacillus fermentum* is associated with advanced lesions due to the metabolic cooperation with *S. mutans* [1]. Cariogenic biofilm has lower diversity of microbes than non-cariogenic biofilms [1]. In most cases, how the microorganisms are spatially organized with each other in the dental biofilm to promote disease remains unknown [17]. The presence of specific functional ecotypes with a propensity for saccharification or proteolysis may be a driver of oral biofilm disease.

#### 2.1.4. Scardovia Species

Recent evidence has begun to reveal new caries-related microbes that may alter the current understanding of the pathobiology of dental caries. *Scardovia Wiggsiae* (*S. wiggsiae*) is a gram-positive anaerobic bacterium with high acid production and acid resistance properties. It is ecologically competitive in acidic environments and considered a potential cariogenic bacterium. Although the carious mechanism of *S. wiggsiae* is not fully understood, its correlation with early childhood caries (ECC) has been confirmed by some scholars. Kameda et al., (2020) demonstrated that the F6PPK shunt metabolic pathway of *S. wiggsiae* may result in high lactic acid tolerance and thus renders it ecologically competitive in the cariogenic biofilm microenvironment [18]. Matondkar et al., (2020) found that the presence of *S. wiggsiae* in children’s plaque was significantly correlated with ECC [19]. Contrary to these studies, Inquimbert et al., (2019) indicated that *S. wiggsiae* was highly expressed in both caries and caries-free subjects [14]. Therefore, whether *S. wiggsiae* can be classified as cariogenic bacteria is still unclear and further research is required.

#### 2.1.5. Other Species

Previous studies compared the oral microbiota between adolescents with high prevalence and low prevalence of caries. Sequencing-based results showed a high abundance of *Actinomyces* and *Prevotella* in the dental biofilm from caries-active adolescents [17]. The available data tends to imply that the *Candida* species correlates with dental caries [20,21]. The overexpressed collagenases for proteolytic metabolism in the *Prevotella* species may lead to the progression of dental caries. The fimbriae of *Actinomyces viscosus* (*A. viscosus*) has a strong affinity for the collagen in the cementum, which promotes the attachment onto the surface of hydroxyapatite. Moreover, the co-culture of *Candida albicans* and *A. viscosus* can produce more polysaccharides as a metabolic substrate, which provides energy and prolongs acid production time [22].

However, it is not the microorganisms themselves but the microenvironments created by the above microorganisms that increase the risk of dental caries. In contrast, we believe that the process of the above cariogenic microorganisms involved in the formation of a cariogenic ecosystem is worth more exploration and the interspecies interactions play important roles in the establishment of the cariogenic biofilm [23].

### 2.2. Biofilm Microbiota: Interspecies Interactions

With respect to cariology, certain interspecies interactions in biofilm may interfere with cariogenic microbes to slow the disease progression, whereas others can mediate ecological dysbiosis to assemble a cariogenic biofilm and/or other oral diseases. The interactions of oral microbes occurring within dental biofilms include physical, nutritional synergy, antagonism, quorum sensing, and gene transfer [24]. Initially, planktonic cells are anchored to the surface of enamels via specific adhesion receptor mechanisms in mature acquired pellicles [25]. Early colonizers that adhered to the surface of teeth can communicate metabolically and promote mutualistic community development, while middle and late colonizers may interact cooperatively or competitively [24,26]. During the development of the cariogenic biofilm, certain microbial interactions can play key roles in low pH environment assembly. Bacteria such as *S. mutans* can reset the microenvironment for other aciduric-cariogenic bacteria to thrive and become established.

Dental biofilm microbes interact through multiple pathways. Host proteins and saliva glycoproteins are the primary nutrients for dental biofilm microbes. The catabolism of glycoproteins requires the action of mixtures of glycosidases and peptidases. However, oral microbes express glycosidases with different specificities and individual bacteria, therefore, depend on the metabolic capacity of other species in order to obtain essential nutrients [24]. Evidence is emerging that oral commensals may affect the acid production, protein synthesis, and antimicrobial molecules release of glycometabolic microbes. Chen et al., reported that co-culture with *S. gordonii* can decrease the mucin production of *S. mutans* and increase the expression of two manganese transporter operons (slo and mntH) [27]. Communication of oral microbes that occur within the biofilm may rely on the signaling molecules. Quorum sensing (QS) mediated by competence stimulating peptide (CSP) [28], sigma X, and autoinducer 2 [29] showed differential effects on biofilm formation. The amount of 21-CSP secreted by *S. mutans* positively correlated with its population density during the process of QS to initiate biofilm formation and bacteriocin production. Meanwhile, signaling molecules may help recipient cells in taking up extracellular DNA while gene transfer tends to make bacteria more resistant to the harsh condition. The autoinducer-2 (AI-2) induces the ability of the microorganisms in the community to tolerate acid and oxidative stress and to form biofilms [30]. The origin of the Streptococcus interspecies genes that confer penicillin resistance could be identified in oral streptococci and this evidence represents interspecies genetic exchange [31]. Altogether, a pathological process relies on the interspecific interactions and those interactions in the cariogenic biofilm have not been fully understood.

### 2.3. Matrixome in Cariogenic Biofilms

Biofilm is not just the sum of microbes as matrixome provides an essential scaffold for the existence of a biofilm lifestyle and the expression of a pathogens’ virulence. It is still not clear if caries begin with bacterial succession to become pathogenic to form cariogenic biofilm or if the change of components in dental biofilm results in a shift from being a harmless biofilm to a harmful one. Biochemical properties of the matrix play a key role in the determination of biofilm cariogenic risk. Bowen et al., (2018) emphasized that the caries process is affected by virulence attributes of biofilm matrixome [1]. At the initial stages of the dental biofilm, the extracellular polymeric substances (EPS) matrix provides biofilm surface adhesion properties and the adhesion and cohesion of the EPS matrix in a cariogenic biofilm are higher when compared to those in harmless biofilm [1]. The structural and spatial organization of EPS provide cariogenic biofilm antimicrobial recalcitrance. In addition to providing mechanical stability, the matrix causes microenvironment gradients to form and permits the organization of cariogenic pathogens into cohesive multicellular ecosystems, which creates low pH localized niches [1,32]. Buffer saliva has no access to the acids produced deep in the dental biofilm due to the structure of EPS, while the sugars in the diet can easily diffuse throughout the cariogenic biofilm [33]. The main components of EPS in the cariogenic biofilm are polysaccharides, proteins, nucleic acids, and lipids [34,35]. Host proteins and glycoproteins could also contribute to the adhesion and matrix establishment of the pathogen as a nutrient source for microbes in biofilm. The composition of EPS affects their functions, such as the enhancement of cell-to-cell binding and the maintenance of the acidic microenvironment. Microbes can sense biofilm matrix cues and trigger cellular responses for remodeling their surrounding matrix; this is called cell–matrix interaction. Cell–matrix interactions occurring within cariogenic biofilm could enhance acid-producing signaling and adhesive functions locally. Therefore, there are often local regions of hypoxia and low pH [36]. Targeting matrixome (biofilm-specific condition) could lead to precise approaches against cariogenic biofilm.

## 3. Diagnosis of Cariogenic Biofilm

Molecular biological techniques have led to a breakthrough on the roles of the oral microbiome in the caries process. Researchers concluded on the evidence available that the microbial composition and microenvironment of dental biofilm were transformed before the onset of dental caries lesions [37,38]. Therefore, detection of the cariogenic biofilm would demonstrate a new train of thought on the early diagnosis of dental caries and assessment of the progress rate of caries lesions.

### 3.1. Biomarker of Cariogenicity

Salivary mass-spectrometry and proteomic profiles combined with microbial genomic analysis were deemed as an accurate method for caries risk assessment [39]. Salivary biomolecules could serve as potential biomarkers for cariogenicity of the oral microenvironment. However, it must be noted that salivary components differ depending on gender and age [40,41]. In a recent study, Hemadi et al. (2017) reported that the process of acidification of the oral ecosystem could be indicated by alterations in salivary protein components. They found that the concentration of salivary LL-37 in saliva could indicate the level of *S. mutans* in oral microbiota [40]. β-defensin-2 was detected in high concentrations in caries-active subjects. Therefore, the finding on the alterations in the salivary composition may indicate the formation of localized cariogenic biofilms.

### 3.2. Diagnosis of Biofilm Acidification

**Figure 2:**
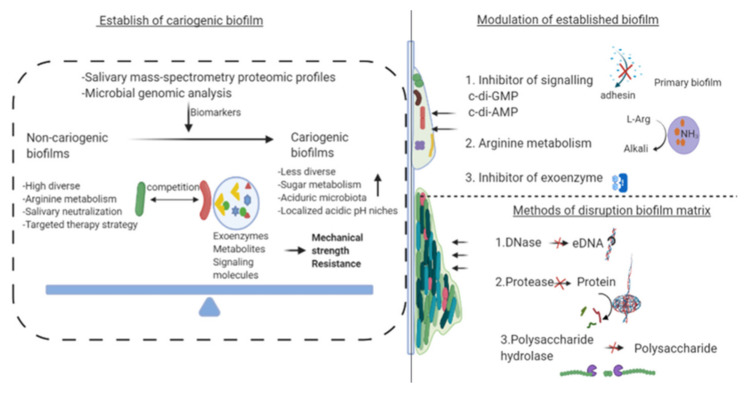
Establishment of cariogenic biofilm and therapeutic strategies targeting the cariogenic biofilm. Competition between oral resident microorganisms and opportunistic pathogens will shift the dynamic balance of dental biofilm microflora. In the cariogenic biofilm, microbial diversity decreases as an aciduric microbiota predominates. The effective therapies to control cariogenic biofilms include the modulation of biofilm microbiota and the disruption of the biofilm matrix. The illustration was created with BioRender.

The acidification of the oral ecosystem is a relatively long process. The dynamics of microbe-microenvironment interrelation within dental biofilm play an important role in biofilm acidification. Competition of the organisms associated with health could interfere with the factors that enable the cariogenic bacteria to destroy the homeostatic mechanisms, such as sugar metabolism (Figure 2). The metabolites of cariogenic microorganisms in the presence of fermentable sugars, especially sucrose, cause biofilm acidification. Meanwhile, an acidic microenvironment leads to the dominance of aciduric-cariogenic microorganisms, such as *mutans streptococci*, and will increase selectively [42]. Evidence primarily derived from next-generation sequencing suggested a slight and significant modification on microbial diversity between subjects with and without active caries [37,43]. LDA effect size (LEfSe) analysis demonstrated that the decrease in abundance of corynebacterium might be interpreted as a harbinger of biofilm acidification [43]. When Jiang et al. (2014) investigated the shifting of microbial profiles in the progression of dental caries, they found that *Cryptobacterium*, *Lactobacillus*, *Megasphaera*, *Olsenella*, *Scardovia*, *Shuttleworthia*, *Cryptobacterium*, and *Streptococcus* were increased significantly in cavitated dentin lesions [37]. Clinical research carried by Liu et al., (2019) found that mutans streptococci and lactobacilli levels were high in cariogenic biofilm due to their capability of acidogenesis at low pH [44].

## 4. Recent Advances in Inhibition of Cariogenic Biofilms

Traditional therapies for preventing dental caries include dental caring education, diet counseling, and the chemical and mechanical control of cariogenic biofilms. Although most of these methods, such as the application of fluoride and antibacterial drugs, are effective and economical, behavioral and cultural factors may limit these therapies in different individuals or populations. We outlined recent advances in cariogenic biofilms modulation presented in academic publication databases (Table 1).

### 4.1. Effect on Bacterial Diversity of Cariogenic Biofilm

**Figure 3:**
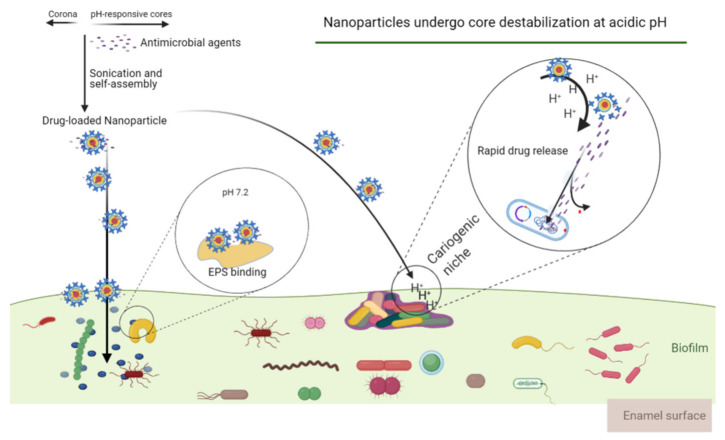
pH-triggered release of nanoparticles and their effect on cariogenic biofilm. Nanoparticles would bind to pellicle and EPS surfaces at physiological pH. Acidic pH is employed as a trigger for micelle destabilization and rapid drug release in the cariogenic niche. As cariogenic biofilm microenvironments may reach a pH of 4.5–5.5, therefore, pH-activated nanoparticles rapidly release bioactive agents to enhance drug retention at at-risk sites. The illustration was created with BioRender.

Although there are broad-spectrum antimicrobials with high efficacy, they could disrupt the ecology of oral microbiota. There are currently no therapies that specifically target cariogenic microorganisms and yet do not disturb the overall oral microbiome. As people gradually realize the importance of maintaining oral microecology, the exploration of compounds that specifically inhibit pathogens is in full swing. *S. mutans* contributes to caries pathogenesis [30]. As early as 2006, Eckert et al. designed antimicrobial peptides (denoted C16G2) that specifically inhibited *S. mutans*. C16G2 could mediate *S. mutans*-specific delivery of antibacterial agents [63]. Tian et al. (2017) identified a new peptide HP30 after evaluating several fusion peptides and reported that the specific killing activity of HP30 relied on the ComD receptor of *S. mutans* [64]. C10-KKWW was identified and selected by Xiang et al. (2019) and was proved to break down the cell membrane of *S. mutans*. Moreover, they found that C10-KKWW could target L-ascorbate translocation to eradicate *S. mutans* [65]. Precision therapy is valuable research in the field of cariogenic biofilm prevention and could help the reengineering of oral microbiota [66]. The application of nanoparticle strategies could provide unparalleled flexibility for the time point and niche of drug release, especially for cariogenic microorganism treatment, exploiting acidic microenvironments for stimuli-responsive drug release [67]. Furthermore, nanoparticles can pass the biofilm matrix and benefit drug delivery (Figure 3). Naha et al., (2019) reported that dextran-coated iron oxide nanoparticles are activated by acidic biofilm microenvironment to produce hydrogen peroxide at acidic pH niche thereby displaying strong local antibacterial activity without adverse effects on the oral microbiome [45]. Similarly, Huang et al. (2021) exploited a functional nanozyme that could cause localized bacterial killing in the cariogenic biofilm. In acidic pH, the iron oxide nanoparticles containing glucose-oxidase can trigger reactive oxygen species production to selectively kill *S. mutans*. The anti-caries activity of nanozyme was also assessed in a rodent model of dental caries and the data revealed that material with short time exposure greatly attenuated enamel demineralization on the tooth surfaces [54]. The specificity and efficacy of nanoparticles on the inhibition of cariogenic biofilm are closely related to their pH-dependent binding affinity and pH-triggered release [68]. Meanwhile, histatin and statherin peptides have been shown to enter the acquired pellicle as antimicrobial peptides to inhibit pathogen adhesion [69]. However, the above studies were simply controlling cariogenic bacteria without a view on the whole biofilm ecosystem, which might be contra-productive. The formation of cariogenic biofilm is a pathological process that relies not only on microbial composition but also on the expression of their virulence.

### 4.2. Modulating Virulence and Effects on Active Attachment System of Biofilm

Technological advancements have exposed the complexity and importance of the cariogenic microbial community. Biofilm is a highly structured community in which cariogenic biofilm is recognized as an essential virulence factor associated with dental caries. Early studies focused on inhibiting the pathogens; however, it is now clear that EPS matrix may interfere with the efficacy since it allows the formation of localized acidic niches. Here we summarized the therapeutic strategies targeting the cariogenic biofilm matrix (Figure 2). The matrix not only helps to avoid shear and other stresses but also enhances the tolerance of antimicrobials. Studies have shown that changes in the external environment and even anti-biofilm therapy can activate biofilm-specific adaptation mechanisms, thereby enhancing the integrity of the established biofilm communities. Such changes in the biofilm community structure may play a central role in the notorious obstinacy of biofilm infections [70]. Carbohydrate catabolite repression and the breakdown of EPS are effective methods against cariogenic pathogenicity [65,66,67]. Rainey et al., (2019) demonstrated a new virulence factor of *S. mutans* (glycosyltransferase SMU_833) that modulates the synthesis of glucan and the release of eDNA, thereby altering biofilm matrix constituents [71]. Horev et al., (2015) developed farnesol-loaded nanoparticles that display high EPS matrix degradation activity [72]. Recently, therapeutic strategies targeting the ecological microenvironment and cariogenic biofilm have emerged. Garcia et al. (2017) identified a small molecule with notable selectivity toward *S. mutans* biofilms through screening a library of 2-aminoimidazole antibiofilm compounds. The small molecule (denoted 3F1) worked as a chemical probe and proved that selective targeting therapies are feasible [73].

## 5. Concluding Remarks and Future Perspectives

This article presents dental biofilm and its mechanisms in initiating and aggravating the cariogenic ecosystem. Different modes of action and strategies for modulating the biofilm microenvironment are outlined. The antimicrobial agents listed in Table 1 combined with the technique shown in Figure 3 may offer new ideas to the selective targeting of cariogenic biofilm. The microbial communities involved in cariogenic biofilm display sophisticated structural and functional integration and this concurs with published systematic studies. In addition to focusing on pathogens, the possibility that biofilm microenvironment might also show an equally strong association with caries should be examined. Targeting biofilm-specific conditions, such as the matrixome, could lead to a precise and effective anti-caries approach. It has been hypothesized that oral microbiota dysbiosis is involved in oral cancer [74] and numerous systemic diseases, such as systemic lupus erythematosus [75], Alzheimer’s disease [76], obesity [77], rheumatoid arthritis, chronic obstructive pulmonary disease [78], and diabetes [79]. Recent evidence also supported that oral microbiota dysbiosis is a collaborator in preterm birth and preeclampsia [80]. Therefore, alternative ecological prevention and treatment strategies that focus on restoring health-related ecosystems rather than killing pathogens would be better for creating an environment that is not conducive to the development of caries. These interventions should be able to change acidic environmental factors with limited destruction of beneficial bacterial communities. Generally, it is accepted that the approaches would not affect the resistance of pathogens.

Altogether, understanding the underlying mechanisms of cariogenic biofilm is key to effectively reduce the adverse effects of the current therapeutic strategy [81]. Opportunities for improved efficacy as well as new biomimetic strategies should be carefully considered to ensure that dental caries-related biofilm therapies continue to move forward.

## Figures and Tables

**Table 1:** Recent advances in modulation of cariogenic biofilms.

The Material Used for Biofilm Modulation	Targets	Mechanism	Reference
Dextran-coated iron oxide nanozymes; H_2_O_2_.	Acidogenic biofilm (bacterial killing and EPS-matrix breakdown).	Nanozymes catalytic H_2_O_2_ at acidic conditions.	Naha et al. [45] (2019)
Sonodynamic excitation of nanomicelle curcumin.	*S. mutans* (local therapy).	Curcumin activated by ultrasound waves irradiates and produces the ROS.	Pourhajibagher et al. [46] (2020)
Probiotic.	Composition of cariogenic biofilm.	Modification of inherent ADS activity; Production of antimicrobial agent (bacteriocin and hydrogen peroxide); Metabolism of lactic acid.	Chen et al. [47] (2020)
DMAEM and HMAEM [tertiary amine]	*S. mutans* biofilms; Microbial diversity of saliva-derived biofilms.	Materials have long-term reversible acid-activated properties that could quickly show an antibacterial effect via protonation.	Liang et al. [48] (2020)
TBO-mediated photodynamic therapy.	Cariogenic biofilms.	TBO can absorb light energy and catalyze the formation of ROS.	Balhaddad et al. [49] (2020)
Peptide GH12.	Acidogenic bacteria.	Net positive charge of GH12 increased and the tryptophan fluorescence intensity heightened with the peak shifting towards the short wavelength at pH 5.5, which demonstrated that GH12 could be more easily attracted to the anionic microbial cell membranes and that GH12 showed stronger interactions with the lipid membranes.	Jiang et al. [50] (2020)
Biosurfactant; Chitosan.	Cariogenic microorganisms.	The surfactant can associate strongly to the polymer, which generally leads to the occurrence of micellisation at lower concentrations of the tensioactive agent; Chitosan chain (NH^3+^) positive charges and the negatively charged cell wall and/or cytoplasm membrane of the microbial surface cause the breakdown of these structures and the loss of intracellular material.	Farias et al. [51] (2019)
Photodynamic inactivation employing methylene blue with irradiation from a red laser.	*S. mutans* biofilms.	High quantum yield (ΦΔ ≈ 0.5) and long absorption wavelength (λmax = 664 nm; red light), which allows better light penetration in live tissue.	Legéňová et al. [52] (2020)
Curcuma xanthorrhiza nanoemulsions.	*S. mutans* biofilms.	For nanoemulsions with nano-sized droplets stability can be maintained for a long period of time because their diffusion rate is higher than gravity settling or creaming due to Brownian motion; the antimicrobial activity is mainly attributed to the –OH group and the hydrocarbon chain of xanthorrhizol.	Cho et al. [53] (2020)
Bi-functional nanozyme.	Cariogenic biofilm microenvironment.	The nanohybrid contains glucose-oxidase that catalyzes glucose present in biofilms to increase intrinsic H_2_O_2_, which is converted by iron oxide nanoparticles with peroxidase-like activity into ROS in acidic pH.	Huang et al. [54] (2021)
Napabucasin.	Oral streptococcal biofilms.	Napabucasin exhibited good antimicrobial activity against oral streptococcal planktonic cultures and biofilms but with lessened cytotoxicity as compared to chlorhexidine.	Kuang et al. [55] (2020)
Propolis.	Dental plaque in the mouth of high caries risk children.	Propolis as a natural product has high bactericidal effect and low toxicity.	El-Allaky et al. [56] (2020)
Silver diamine fluoride.	Cariogenic bacteria isolated from human saliva.	Electrostatic adhesion of silver ions with bacterial enzymes inactivates them and prevents metabolic activities of the bacterial enzymes via silver-induced protein coagulation; fluoride inhibits demineralization by being absorbed onto the hydroxyapatite crystals and are resilient to a repeated acid attack; silver and fluoride shows synergistic effects.	Sorkhdini et al. [57] (2020)
Chlorophyllin-phycocyanin mixture.	*S. mutans* biofilms.	The decrease in metabolic activity can be due to the 8-fold to 10-fold increase in the production of ROS in the photodynamic process that by reducing the membrane potential and intracellular adenosine triphosphate affects cell membranes.	Afrasiabi et al. [58] (2020)
*Allium sativum* bulb.	Cariogenic biofilm.	Allicin showed high antibacterial activity against the cariogenic bacteria due to protease inhibiting ability.	Bin et al. [59] (2020)
Psidium sp., Mangifera sp., Mentha sp., and its mixture,	Cell-surface hydrophobicity; initial pH change in the oral biofilm; *S. mutans* adhesins.	The phenolic content of the plant extracts may interfere with the adhesion of bacterial cells in the experimental pellicle; the plant extracts create a balance between the two bacterial species.	Shafiei et al. [60] (2020)
Zein nanoparticles containing anacardic acid.	*S. mutans* biofilms.	The activity of the inhibit bacterial proliferation of anacardic acid was associated with the ability to permeate the lipid bilayer of cell membranes and causes its disruption; nanoparticles from corn protein zein that are biodegradable and have a relatively low cost provides anacardic acid stabilization and enhanced its esthetic characteristics.	Lima et al. [61] (2020)
Antimicrobial peptides derived from eutericin 6 and gassericin A.	*S. mutans* biofilms.	Selective membrane disruption.	Liang et al. [62] (2020)

H_2_O_2_: Hydrogen peroxide; *S. mutans*: *Streptococcus mutans*; ROS: reactive oxygen species; ADS: arginine deiminase system; DMAEM: dodecylmethylaminoethyl methacrylate; HMAEM: hexadecylmethylaminoethyl methacrylate; TBO: toluidine blue O.
